# One country's journey to interoperability: Tanzania's experience developing and implementing a national health information exchange

**DOI:** 10.1186/s12911-021-01499-6

**Published:** 2021-04-29

**Authors:** Alpha Nsaghurwe, Vikas Dwivedi, Walter Ndesanjo, Haji Bamsi, Moses Busiga, Edwin Nyella, Japhet Victor Massawe, Dasha Smith, Kate Onyejekwe, Jonathan Metzger, Patricia Taylor

**Affiliations:** 1 USAID’s Maternal and Child Survival Program/John Snow Inc., Dar es Salam, Tanzania; 2 USAID’s Maternal and Child Survival Program/John Snow Inc., Arlington, USA; 3 Information, Communication and Technology (ICT) Unit, Ministry of Health, Community Development, Gender, Elderly and Children (MOHCDGEC), Dar es Salam, Tanzania; 4 USAID, Health System Strengthening, Dar es Salam, Tanzania; 5 Center for Digital Health, John Snow Inc., Arlington, USA

**Keywords:** Health, Interoperability, Standards, Architecture, Governance

## Abstract

**Background:**

Robust, flexible, and integrated health information (HIS) systems are essential to achieving national and international goals in health and development. Such systems are still uncommon in most low and middle income countries. This article describes a first-phase activity in Tanzania to integrate the country’s vertical health management information system with the help of an interoperability layer that enables cross-program data exchange.

**Methods:**

From 2014 to 2019, the Tanzanian government and partners implemented a five-step procedure based on the “Mind the GAPS” (governance, architecture, program management, and standards) framework and using both proprietary and open-source tools. In collaboration with multiple stakeholders, the team developed the system to address major data challenges via four fully documented “use case scenarios” addressing data exchange among hospitals, between services and the supply chain, across digital data systems, and within the supply chain reporting system. This work included developing the architecture for health system data exchange, putting a middleware interoperability layer in place to facilitate the exchange, and training to support use of the system and the data it generates.

**Results:**

Tanzania successfully completed the five-step procedure for all four use cases. Data exchange is currently enabled among 15 separate information systems, and has resulted in improved data availability and significant time savings. The government has adopted the health information exchange within the national strategy for health care information, and the system is being operated and managed by Tanzanian officials.

**Conclusion:**

Developing an integrated HIS requires a significant time investment; but ultimately benefit both programs and patients. Tanzania’s experience may interest countries that are developing their HIS programs.

## Background

Achieving the Sustainable Development Goals (SDGs) espoused by the United Nations General Assembly will require stronger, well-financed and better-staffed health systems, with foundational elements that include dynamic health information systems (HIS) to monitor progress in the sector. Tanzania’s Health Sector Strategic Plan points to the need for a stronger HIS, one that supports data exchange and interoperability—defined as the capacity within the system to share and use data from two or more systems to improve its use at all levels of the health system [1, 2]. These improvements align with the World Health Organization’s (WHO) framing of HIS as a fundamental health system building block and promotion of reliable, timely data to inform policy, program and individual decision-making, and guide the efficient distribution of resources [3–5].

Optimally, a health information system includes data about the health workforce, financial systems, client visits, commodities, disease surveillance, vital registration data, and other health-related information at facility and community levels to facilitate planning, identify gaps, support decision-making, and prioritize resources [2, 6]. Data from these often separate systems are collected and managed through multiple forms, using digital and/or paper tools, across public and private health systems. The data from these systems are often collected and stored using different protocols and formats and, as a result, they are difficult to share or compare.

An interoperability layer that supports data exchange across multiple standalone information systems and domains is a critical tool for unlocking the power of data in increasingly digitized health information systems. Developing an interoperable system and harmonizing a country’s overall health care strategy in a manageable and sustainable manner, requires structured architecture [7, 8]. Enterprise Architecture (EA) is a strategic approach to mapping business functions and existing and future information flows that has been shown to help planners decide how to integrate and share data between different systems and across locations, so that information flows feed into and support a larger, more connected networking environment.

EA is already used by governments and businesses across the globe, and was used in Tanzania to define the national HIS governance and operating structure, optimize and integrate technical applications, network programs and locations, determine needed expansion capacity, and standardize processes [3]. Tanzania also has supported international best practices, such as adhering to and promoting the Principles for Digital Development^1^ while building out its national HIS. EA fit into the country’s plans for developing a comprehensive eHealth system.

This article describes a partnership (2014–2019) between Tanzania’s Ministry of Health, Community Development, Gender, Elderly and Children (MOHCDGEC) and the U.S. Agency for International Development’s (USAID) flagship Maternal and Child Survival Program (MCSP) to develop an integrated, interoperable health information system to improve health outcomes by enabling cross-program data exchange via an interoperability layer. The Ministry led activities on this project, called the Tanzania Health Information Exchange (Tz-HIE) Project, with support from MCSP and other partners.^2^ The HIE is currently operating and is functioning across five health-related data domains, and is being managed by the Information and Communication Technology (ICT) department of the MOHCDGEC. Here, we describe the process that led to the system’s development and early deployment.

### Building Tanzania’s health information system

For more than 15 years, the United Republic of Tanzania has focused on designing and deploying a robust routine HIS that collects and reports data across multiple health system domains. Activities supporting this goal include implementing the District Health Information Software (DHIS2), strengthening infectious disease surveillance, and incorporating human resource management into digital systems [9–14]. The Ministry’s eHealth Strategy for 2013–2018 focused on establishing eHealth standards, rules, and protocols for information exchange and protection, and comprehensive health facility, provider, and client registries. In the Health Sector Strategy Plan for 2015–2020, the MOHCDGEC committed to achieving interoperability and “the rapid development of ICT for improving administrative processes, patient/client recording and communication” [15].

In 2005, the MOHCDGEC began working with the USAID | DELIVER Project and other partners to improve national health information logistics and strengthen human capacity and systems design. This work led to implementation of the Integrated Logistic System Gateway, a mobile reporting system designed to increase the visibility of logistics data and improve product availability, and subsequently the electronic Logistics Management Information System (eLMIS), which increases supply chain visibility at all system levels [16–18]. Other routine and non-routine health information were collected through household surveys (such as the Demographic and Health Surveys), health facility and administrative databases, census and vital events registration data, the Integrated Disease Surveillance and Response framework for communicable disease surveillance, and mobile technology for data collection on immunizations and neglected tropical diseases [19–22].

Over the past decade, the use of mobile technologies (mHealth), electronic medical records (EMR) at hospitals, medical decision-making tools, and other health-related tracking systems for infectious disease assessment, community-based services delivery, outreach services, and need identification and prioritization has increased [19–22].

However, in Tanzania as elsewhere, these tools have not always linked effectively to the overall HIS data, leading to duplication of work, data quality errors due to manual transmission, and inappropriate use of data. In Tanzania, the lack of electronic data exchange hindered service delivery, and weakened linkages between health information system components [23–26]. Limited interoperability also diminished information accessibility, compatibility, and sharing across data sources housed at universities, professional councils, non-governmental organizations, the MOHCDGEC,^3^ and the Ministries of Education and Finance, resulting in chronic data gaps and missed opportunities to use new and promising practices, tools, and approaches.

To address these gaps, the government of Tanzania and its partners examined several global networks that help countries develop common frameworks and design, and implement HIS architecture and interoperability. One such network is AeHIN (Asia eHealth Information Network—https://www.asiaehealthinformationnetwork.org/), which promotes use of ICT in Asia and uses the “Mind the GAPS (**g**overnance, **a**rchitecture, **p**rogram management, and **s**tandards) framework” to support countries. Another community of practice supporting architecture and standards-based interoperability in health sector is OpenHIE (Open Health Information Exchange—https://ohie.org/) which has developed various resources around HIS architecture, system functionality, and standards, and provides access to various open-source digital solutions that are available for countries to adopt and learn.

**Fig. 1:**
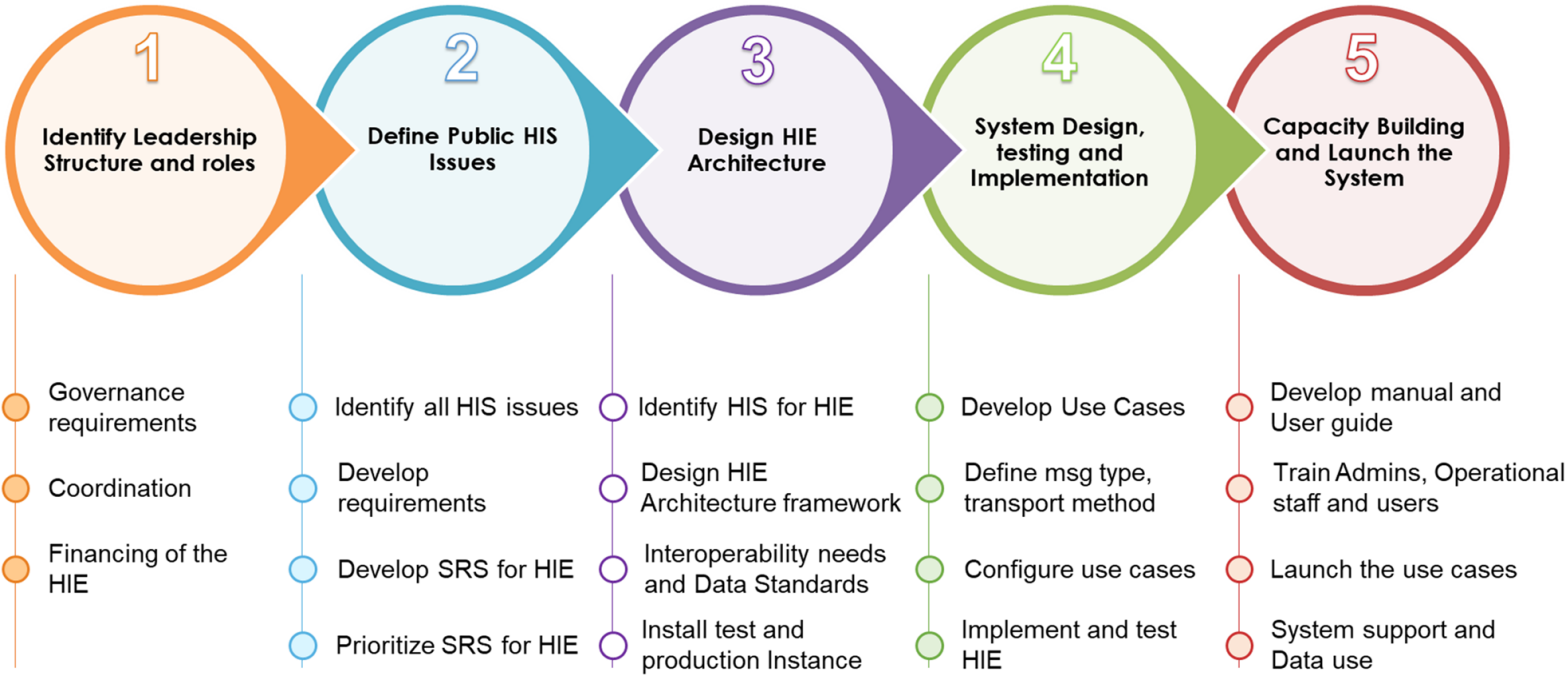
Tanzania HIE implementation approach

To move toward interoperability throughout the health care system, the MOHCDGEC employed AeHIN’s five-step approach. These enabled the incorporation of an interoperability layer into the development and implementation of an integrated health information exchange (TZ-HIE). The work in Tanzania also emphasized two more priorities—improving data use, and capacity building for sustainability adopting a GAPS-CU (capacity and use) approach (see in Fig. 1 and details in subsequent text):

1. Identifying leadership structures and roles
2. Defining public health information system priorities
3. Designing the HIE architecture
4. Designing, testing, and implementing the system
5. Building capacity and supporting data use

As part of Phase I implementation, the MOHCDGEC prioritized four use cases to demonstrate how interoperability would work (described in Step 2). This article focuses describes the process by focusing on the first use case—improving access to and use of data across specialized hospitals. Please note: use cases are specific to the needs of each country / each Ministry of Health. The implementing organization deferred to the priorities established by the Government of Tanzania. Accordingly, readers should infer that the methodologies employed in the Tanzania study are generalizable because it is less about the specifics of the use-case, and more about how to address system-level change. Accordingly, while the first use case is discussed below, please note that the Ministry’s approach followed the same five GAPS-CU steps for design and implementation of each case.

## Methods: the five-step approach for developing the HIE

### Step 1: identifying leadership structures and roles

#### Determining leadership and structure

The Broadband Commission’s Working Group on Digital Health states that, “strong leadership, intersectoral collaboration and clear governance are essential for effective implementation of a national digital health strategy.”^4^ To develop a harmonized overall health information system in Tanzania, it was crucial to identify the leadership and governance structure for the HIE, including coordination, partnerships, and financing. MCSP’s primary activity was to support a Ministry-led process that brought stakeholders together to review key concepts and global models, review national applications, develop a common vision, and determine high-level requirements for a comprehensive HIE framework.

**Fig. 2:**
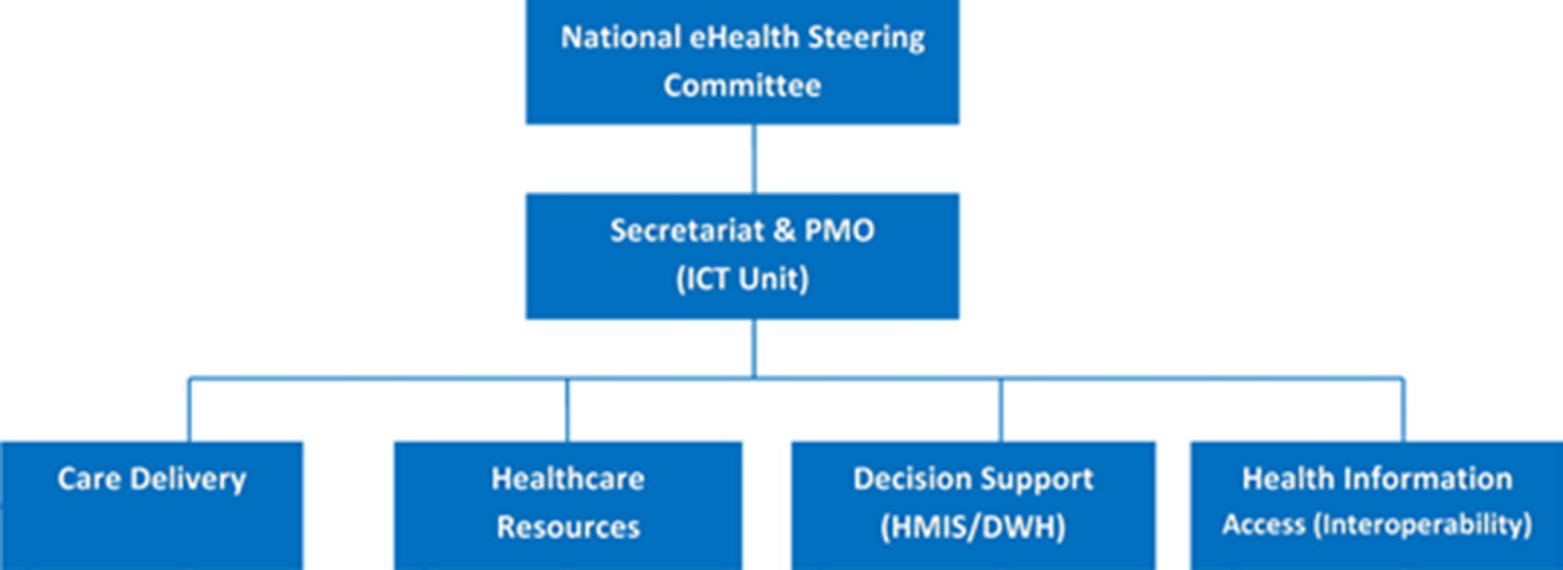
Health information exchange governance structure

Through these discussions, the MOHCDGEC and its development partners defined the high-level data exchange framework that leverages digital health technology to improve key aspects of the health system; achieve the strategic objectives of the Fourth Health Sector Strategic Plan (HSSP-IV); and identify the eHealth system’s architecture objectives, priorities, and gaps. A governance structure was established (Fig. 2) under the leadership of the Permanent Secretary of the MOHCDGEC as the chair of the National eHealth Steering Committee. The Ministry’s ICT unit served as the secretariat, and a Project Management Office (PMO) was formed to coordinate development of multiple elements of the HIS. Under the PMO, four technical working groups (TWGs) were created: care delivery, health care resources, decision support, and interoperability. More than 25 government subject-matter experts from national hospitals, partners, and local and regional government offices took part in the development process and participated in a range of steering committees and working groups.

#### Fostering discussion and education

**Fig. 3:**
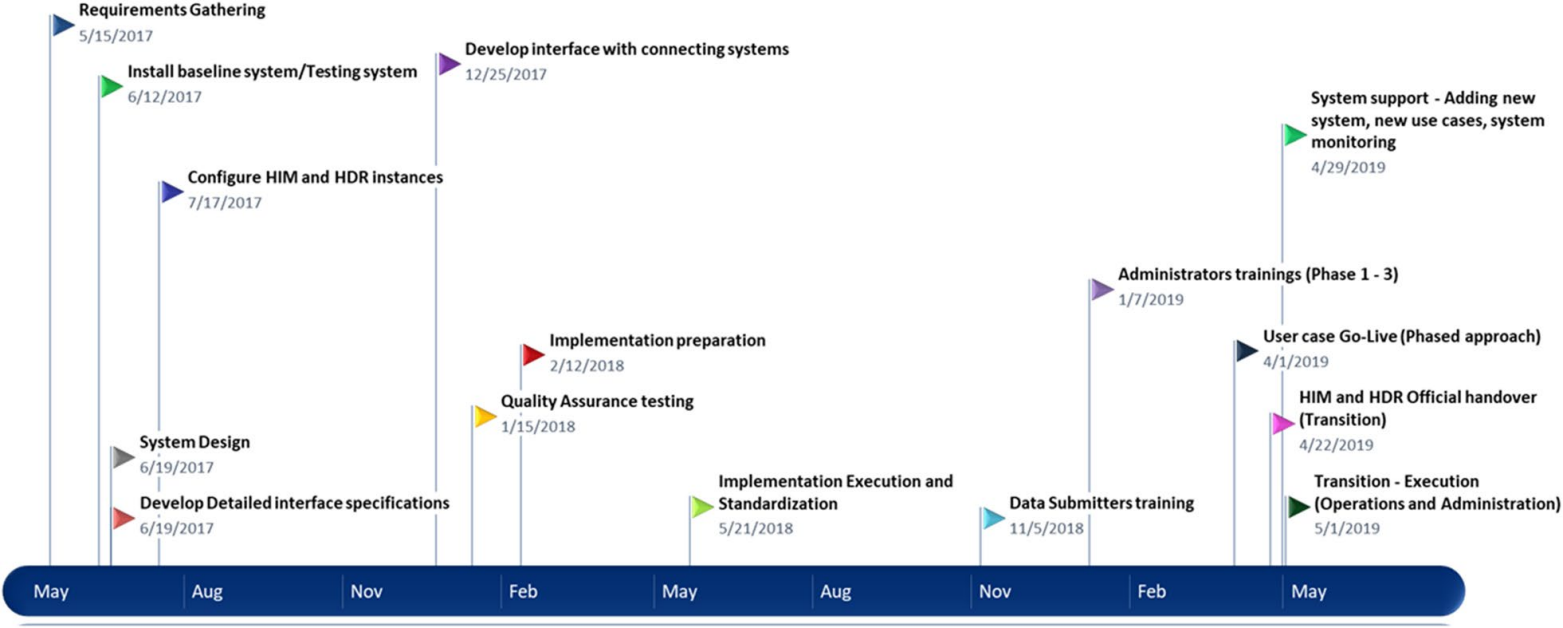
Tanzania HIE phase one implementation timeline

To facilitate discussion among the partners and stakeholders, the Ministry held a series of meetings and workshops including members from the Government of Tanzania including universities such as the University of Dar es Salaam, donors such as USAID and the Gates Foundation, implementing partners such as PATH, JSI, and local organizations. Members were included because of their knowledge of developing information systems, deployment of digital health tools, and policy enabling/enforcing measures. The process included review of case studies to examine examples used by other projects and countries, and brainstorming sessions to identify issues and understand key concepts (e.g., measuring change). After intense discussions, participants voted on priorities and approaches, and drivers and challenges were addressed at multiple points in the process. Detailed timelines are described in Fig. 3. Among the mechanisms used were:

- A National eHealth Steering Committee to review and give feedback on the Tz-HIE Project charter. The Steering Committee had a total of seven members.
- Tz-HIE TWG was created to review and provide feedback on the requirements specifications.
- Various stakeholder workshops were held on use cases, Requirements Specifications solicitation, governance principles, acquisition strategy, and development.
- A high-level stakeholder meeting was held and officiated by the Permanent Secretary of the MOHCDGEC.

#### Clarifying stakeholders’ roles

Coordinating participating organizations^5^ within Tanzania’s eHealth system also required an understanding and alignment of their roles, since each organization operated under its own legal, technical, and political parameters. A situational analysis was conducted to determine the legal and technical environment, governance, data standards, and systems within each participating organization. Decision-makers in some organizations were hesitant about participating, and it was vital to hold meetings with organizational leaders and staff to determine logistics and partner commitments—timelines, legal implications, implications for information technology, and roles and responsibilities.

The Permanent Secretary for Health led the process, which helped to reaffirm the government’s commitment to support the Tz-HIE and improve the country’s overall HIS development. This proactive approach to engagement enabled the MOHCDGEC and partners to increase their ownership of and capacity to manage the Tz-HIE.

### Step 2: defining public HIS priorities

Once the governance structure is in place, the TWGs focused on the second step to identify the biggest data challenges that could be addressed via interoperability—development of a “middleware” function (see further details in Step 3 below). This brought together senior Ministry officials and partners to identify, prioritize, and develop use cases, including detailed documentation of requirements and specifications (Box 1); gather input from stakeholders; customize and configure the system; and test the system based on use case specifications.

Based on the prevailing challenges and the Ministry’s immediate needs, the project team engaged specific stakeholder groups to develop four major use cases to enable:

1. Client-level data exchange for priority hospitals, especially national and specialized hospitals. It has been difficult obtain data from these facilities, since they do not report through DHIS2. There was a need to track the performance in these hospitals on a regular basis, looking at bed occupancy, services delivered, deaths occurring, and revenue collected. Stakeholders for the development of this use case included hospital technical and administrative staff.
2. Aggregate data exchange of commodity data (eLMIS) alongside service delivery data (DHIS2), to enable managers to analyze what medical commodities were consumed and what services were delivered. The stakeholders in this use case included the Medical Store Department (MSD) team, program monitoring and evaluation experts, and the DHIS2 team.
3. Extraction and cross-system sharing of data from the Health Facility Registry (HFR) with other systems (DHIS2, Vaccine Information Management System, electronic Logistics Management Information System, and others)***.*** This was considered important because health facility details are constantly changing from being open, closed or changed to a different grade. It was essential to have a master list of operational facilities that can serve as a source of truth and can update other systems of any changes on the status of the facilities.
4. Facilitating the exchange of health commodities stock status from Medical Store Department Epicor 9 to eLMIS, to ensure that supplies are available when needed throughout the system.

Requirements for each of the defined use cases were grouped into functional and system requirements. *Functional requirements* describe what the system should do—such as its ability to exchange client-level data in a single repository, search for records with data quality issues, and so on*. System requirements* describe how the system should perform: this includes functions such as queuing, translating, error messaging, etc.

The team observed several challenges with improving electronic data exchange:

- A variety of digital tools to support various domains in the health sector, e.g., DHIS2, eLMIS, HRHIS, HFR, and others.
- Use of different Electronic Medical Records (EMRs) by public and private health facilities use based on their need and process.
- Non-standardization of data across multiple systems. Few systems have adopted any data standards for recording information.
- Most of the systems e.g. EMRs from Hospitals are missing APIs for integration.
- Non-existence of standardized unique identifiers of individual clients/patients in the Tanzanian health sector.

The needs of each country vary in terms of what HIS interventions are needed. That said, there are common systems to most countries such as logistics management systems to both catalog medical stock/medicines, the movement of said supplies around the country, and the re-ordering of new supplies. Additionally, governments need a registry of health facilities around the country to they can manage health staff, manage supplies, track the state of health in the country (e.g., a simple metric is bed-occupancy rate), etc. Additional “popular” systems globally include Electronic Medical Records so that patient data can be recorded, shared, and analyzed.

### Step 3: designing health information exchange architecture

#### The enterprise architecture approach

The Tanzanian Public Service Management and e-Government Agency of the President’s Office developed the national Enterprise Architecture (EA) Approach, national EA policy standards, guidelines, and an operations manual in 2013 to ensure seamless exchange of health information [27–30]. The Tanzania EA focused on putting the user first, particularly health care workers and data users, to highlight the decisions they make to provide effective care. Consequently, Tanzania’s EA approach required all digital health stakeholders to incorporate interoperability, open standards, flexibility, collaboration, and technology into their designs.

The EA approach helps identify information needs across multiple domains and health sector building blocks [31]. The Ministry of Health, Community Development, Gender, Elderly and Children chose the EA approach to help define and frame data exchange and interoperability across levels and health domains. This approach defines the health sector as an enterprise, in contrast to the domain-specific (i.e., service delivery, supply chain, etc.) traditional approaches to HIS development. EA enables a more harmonized development of HIS and facilitates identification of data exchange needs between domains.

The major focus in this activity was on the application architecture—the behavior and interactions of the applications used within a business or enterprise. At the higher level, the Tanzania HIE model provides a structural conception that describes how components fit to one another to share and exchange information. At lower levels, the HIE requires detailed interactions between various components of service delivery and systems for a specific service delivery area. For example, unique identifiers are required to track continuity of care, integrated care, insurance coverage, referrals, etc. The system architecture “maps” these processes and guides development of a harmonized system.

#### Collaborative decision-making on system elements

Developing this system was time-consuming and required continuous collaboration and feedback. Throughout the five-year process of design and implementation, Tanzania incorporated input from partners and stakeholders, using a collaborative approach to ensure that the system would meet health sector needs, be easy to learn and use, incorporate the principles and products associated with EA and open access, and promote buy-in.

**Fig. 4:**
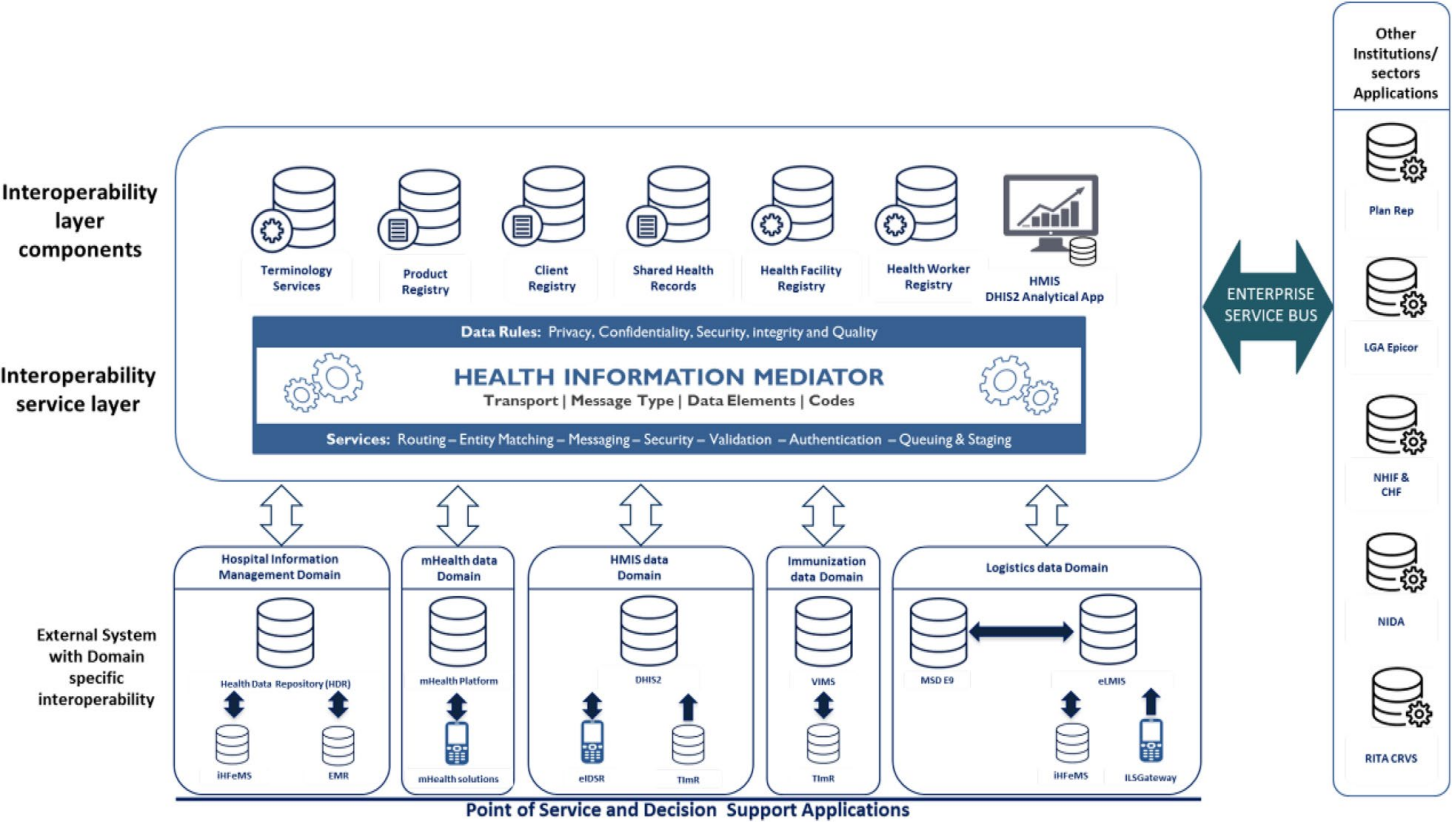
Tanzania HIE blueprint

The National eHealth Steering Committee provided overall leadership and governance for the HIE operation; and the Ministry’s ICT Unit served as the secretariat and management office or PMO. The Tz-HIE blueprint (Fig. 4) represents a dynamic environment that adapts to changing business, information technology, and data requirements.

**Fig. 5:**
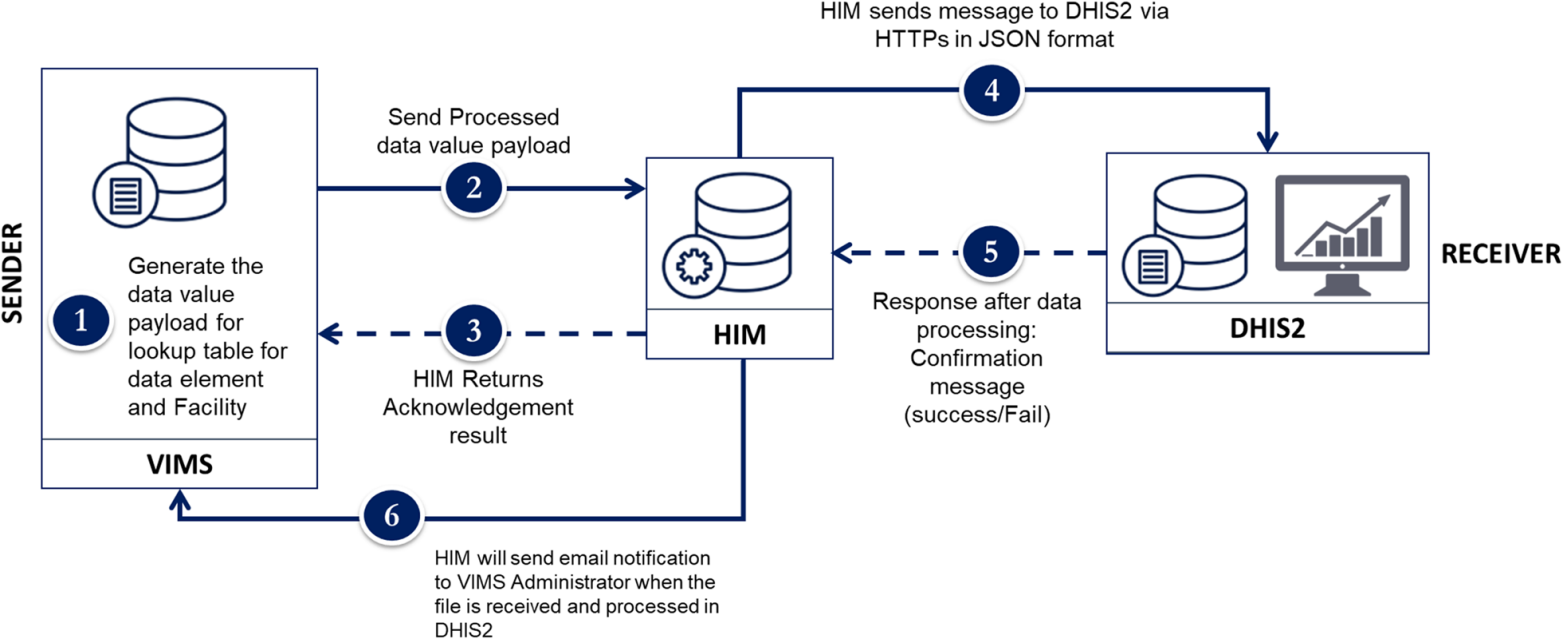
Data architecture for use case 1

The activity in general provided an opportunity to look at the broader health sector-wide need for data and the functions of the proposed data exchange. Once this was done, the next step was to develop use case-specific data exchange architectures and identify components that need to work together. For the purpose of use case 1, the following components of the architecture (Fig. 5) were identified as essential:

1. *Health information mediator *Information sharing and exchange across systems is mediated through a middleware, the Health Information Mediator, or HIM (shown in Fig. 4). A mediator is an essential component of integrated system architecture that facilitates data exchange across multiple systems. It manages functions such as authentication, queuing of messages data translation and data quality check. As of early 2019, the HIM began integrating data across five health domains—Hospital Information Management, mHealth, HMIS, Immunization, and Logistics—each with corresponding sub-domains and their data. HIM implementation addresses the challenges of the point-to-point data exchange by reducing the number of changes that are required to be made to all system connections when one system is modified.
2. *Health data repository or HDR* A database to act as a central repository for all client data collected from multiple hospitals. The repository provides managers access to a real-time from multiple hospitals in a single database.
3. *Terminology services* Houses data standards and data quality protocols and ensures that all transactions are meeting the defined standards and quality protocols.
4. *A dashboard*: To provide visualization and analytical features of performance across multiple health facility activities through the HDR.

The Ministry is now using the HIE conceptual model to align investments and harmonize future development in a national health information system that uses integrated digital technology to provide data for improved decision-making at all levels of the health system.

#### Standardizing data and codes for interoperability

Information sharing through the HIM, and comparison of information across multiple facilities, required harmonization and standardization of service codes. Once the use cases were defined and the architecture designed, the next steps entailed identifying and adopting standards to enable data exchange. For use case 1, a list of data standards were identified, including ICD10 codes for diseases and mortality data and Current Procedure Terminology (CPT) codes for recording procedures. In relation to data standards for use case 1, the PMO team observed that:

1. All hospitals were using ICD10 codes for recording disease and mortality information.
2. Participating organizations had different custom-made service codes, thus rendering it difficult to compare data across systems.
3. Hospitals had different formats for recording dates (e.g., DDMMYY or MMDDYY)
4. Hospitals also used different codes for recording sex and other classifications in their system. (e.g., hospitals recording male, female, others versus M, F, O or 1, 2 and 0)

Table 1 shows the standards used for data exchange; Table 2 shows rules for data processing.

**Table 1:** International data standards used for data exchange

International data standard	Purpose
ICD10	Standardizing data on disease & mortality
CPT4	CPT codes for medical services and revenues such as Evaluation and Management (CPT codes 99201–99499); Anaesthesia (CPT codes 00100–01999; 99100–99140); Surgery (CPT codes 10021–69990) Radiology (CPT codes 70010–79999); Pathology and Laboratory (CPT codes 80047–89398); Medicine (CPT codes 90281–99199; 99500–99607) and Others (CPT codes 0042T—0463T)
Date	YYYY/MM/DD
Sex	M or F

**Table 2:** Sample table showing data processing rules for message and error reports

Data element	Required/optional	Format	Translation rules	Error condition	Indicator for support staff
Message Type	R	Alpha	Must contain SVCREC	If not SVCREC, reject record	Audit report for submitted batch files are available
Org Name	R	Alphanum	Must be registered organization in HIM (tier 2)	If blank or invalid, reject record	Audit report for submitted batch files are available
Local Org ID	R	Num	Must be registered organization in HIM (tier 2)	If blank or invalid, reject record	See above
Dept Name	R	Alphanum	Must be registered organization in HIM (tier 3)	If invalid, reject record	See above
Dept ID	R	Num	Must be registered organization in HIM (tier 3)	If invalid, reject record	See above
Pat ID	R	Alphanum	Patient ID must be alphanum format	If blank, reject record	See above
Gender	R	Alpha	Required. All records must contain gender. Translate as required to match internal gender format	If blank or invalid format, reject record	See above
DOB	R	yyyymmdd	Required	If provided and invalid date format, reject record	See above
Med svcs code	R	Alphanum	N/A	If field blank, reject record	See above
ICD code	O	Alphanum	N/A	N/A	See above
Service date	C	yyyymmdd	Either visit date (for outpatients) or service date (for inpatients) must be provided	If service date is blank, reject the record	See above

Addressing the challenge of non-standard codes for procedures required bringing together health care professionals—including clinicians, radiologists, lab technicians, anesthesiologists, surgeons, cardiologists, physiotherapists, and others—who developed a list of services to be standardized across the country. A standard list of services was defined using the CPT 4 codes from the American Medical Association. These codes were customized for Tanzania and integrated in the terminology services to facilitate data exchange. The list was then mapped against the custom codes used by the hospitals. A digital crosswalk was developed to map the custom hospital codes with the central standard codes, which would enable data from the customized system to the central system. If an exact match was not found, a code nearest to the central code was assigned (Table 3).

**Table 3:** Matching custom hospital codes with CPT4 for standardization

Custom codes used in Hospital A	Custom codes used in Hospital B	CPT code	CPT description	CPT category
LAB13	3042	82945	Glucose (sugar) level on body fluid (CSF,ASCITIC, PERITONIAL,PLEURAL)	5
LAB15	2774	82947	Fasting Blood glucose (sugar) level (FBG) (Blood glucose by strips)	5
LAB21	NA	82951	Blood glucose (sugar) tolerance test (glucose tolerant test-GTT)	5
LAB20	706	82977	Glutamyl transferase (liver enzyme) level (GGT)	5
LAB56	828	83001	Gonadotropin, follicle stimulating (reproductive hormone) level (FSH- FOLLICAL)	5

The HIM, or mediator, incorporated a degree of flexibility to enable alignment with all the systems. For recording dates, if one system recorded client data using the date/month/year system, and another recorded these data using the month/date/year system, the HIM would always translate those systems into the HIE’s operating system.

Data standards are critical for seamless interoperability. However, during implementation, the PMO decided that as an immediate step all custom codes from hospitals would be mapped to the existing CPT code (as shown in Table 3). This was an advantage for the HIM; mandating the use of standards for legacy systems would have been much more challenging, as the systems were already operational and health workers were used to using the custom codes. Over the long term, any new systems being developed will use the standards adopted by the MOHCDGEC, and if there is an opportunity, legacy systems will migrate to using the standardized codes. Introducing new standards did not force organizations to switch, because the flexible interoperability layer translated data from custom to standard code sets.

#### Developing guidance on using the interoperable system

To address emerging issues in the future, the MOHCDGEC developed standards, policy guidelines, and a conceptual framework to organize partners and other stakeholders using digital technology in health care. This approach provided the MOHCDGEC with a complete picture of activities to mobilize and commit resources for specific activities. By late 2017, development of integrated guidelines for facilities using electronic management information systems—guidelines explaining how to use the HIE—allowed the Ministry to assess and strengthen 9 hospital management information systems from 33 national, specialized, and regional hospitals. The conceptual framework, standards, and policy guidelines were each critical to enhancing the system’s ability to integrate data.

#### Step 4: designing, testing, and implementing the system

The next step was to customize the system and conduct conformance testing. Once each use case was prioritized and architecture and interoperability needs identified (steps 1 and 2), the PMO team reviewed and identified an interoperability layer that would fit the Ministry’s needs. Multiple options on the market were already being used to support interoperability in various settings—such as OpenHIM,^6^ OpenFN,^7^ HEALTHeLINK,^8^ mulesoft,^9^ and many others. The PMO team reviewed the performance of various interoperability layer tools against a variety of use cases. The vendors of the tools were given the use case scenarios and asked to demonstrate their tool’s functionalities and ability to manage the proposed use cases.

This was a crucial stage in the journey toward improving interoperability in the Tanzanian health sector. The PMO, which consisted of representatives from multiple organizations and experts, played the role of an independent advisory group. The group reviewed the available tools and advised the MOHCDGEC team to adopt the HEALTHeLINK tool (version 3), which has been used in the U.S. for the past 13 years. This tool, developed using open-source systems such as Linux, Apache, MySQL, and Java, supports data exchange for client-level and aggregate data exchanges in several states. HEALTHeLINK is flexible and provides multiple options to connect with other systems, including APIs, SFTP, and web uploads. It supports interactions with both open source and proprietary systems. This was a key requirement for the health sector in Tanzania, since the national and specialty hospitals (included in the current use cases) were all using proprietary EMRs from external vendors.

With support from MCSP, the team of software developers from HEALTHeLINK worked with the MOHCDGEC and partner organizations to configure the system based on the rules and requirements outlined. The system was then taken through conformance testing to ensure that all requirements were being met and that the system could manage all transactions.

The team found that some systems were unable to participate fully on the HIE, as they were underdeveloped or used outdated technologies that do not support interoperability. To address this challenge, the system was configured to accept data through file uploads (e.g., export/import of XLS files) to the HIE, providing a flexibility that is particularly important in low-resource countries to accommodate key functions. This was a key functionality offered by the chosen tool, HEALTHeLINK.

#### Tools for users

Comprehensive support tools and structures were developed to ensure that the system ran smoothly and supported sustainability. The HIE’s sustainability depends on its flexibility and scalability, as seen in its ability to easily accommodate new use cases or extend existing use cases to cover more organizations. Tools developed for Tanzania included a systems installation manual, users’ operationalization guide, and system administration and implementation guides. The implementation guide was based on firsthand experiences from the Tanzanian context, and provides insight on the HIE processes and best practices to follow when “on-boarding” or connecting new or existing organizations or systems to the HIE.

#### Privacy and security of the System

The system provides the following security features:

- The system provides an audit tool that allows the system administrator to display and print all configuration activities and a report of a current source code audit against security threats.
- Provide alert/notification of security breach.
- The system requires each user to be authenticated by role before gaining access to system.
- Provide encrypted communication between components. Supports encryption of data in flight (when not on a physically secured network) and at rest (whenever data is stored, e.g. when transaction are stored for logging)

### Step 5: building capacity and supporting data use

#### Capacity building

Training and capacity building for both technical staff and users took place throughout the implementation of Tz-HIE. The Ministry used a structured training methodology and standardized training materials for different groups of users. The materials were both adapted from earlier materials used in prior deployments as well as created anew by the software developers supplemented by design specialists who validated the training materials prior to deployment. The training methodology was predominantly hands-on, where participants were given access to the system, with facilitator-led demonstrations and presentations, group assignments, pre-tests, quizzes, and post-tests meant to cement users’ understanding and assimilation of the key issues. The methodology followed the “see-one, do-one, teach-one” approach with the technical specialists to ensure that they knew the material. This was observed by the original designer of the system supplemented by staff who were trained to support. Trainees were from cross Government of Tanzania facilities ranging from the Ministry of Health to Government owned hospitals and universities. To further enhance use of the system, the Ministry’s ICT department provides three tiers/levels of escalated support structure. Level 1 is the technical support team for users at the health facility level. Level 2 is the operations team; and Level 3 targets system administrators.

#### On-the-job support

MCSP also provided technical support to the ICT department by placing a full-time advisor seconded to the ICT unit. The Advisor’s role was to provide technical support the ICT staff and ensure the eHealth Strategy initiatives are implemented as planned. The seconded advisor role included improving coordination across the development of the various eHealth initiatives (including design and implementation of the mediator), identifying the use cases, engaging different departments of the MOHCDGEC, and playing the role of a secretariat for the digital health initiatives in the health sector.

#### Global conferences and meetings

MOHCDGEC officials were supported to take part in various international conferences and study tours—such as the annual Global Digital Health Forum in Washington, DC, the Public Health Informatics (PHI) Conference in Atlanta, GA, and a 2016 HIS study tour in Boston, Massachusetts. The PHI conference provided an opportunity to learn from experiences with interoperability across multiple programmatic area and geographic states in the U.S. The study tour in Boston enabled the MOHCDGEC team to interact with the Massachusetts Department of Health team and learn why and how they are investing in interoperability, and how they use data to make programmatic decisions.

#### Data visualization

To foster improvements in use of the available data by the MOHCDGEC leadership team, dashboards were developed to summarize the data and create insights. These were then displayed on wall-mounted TV screens. The system was set to update the dashboards as new data were sent from the hospitals. The dashboards are also available via a web-link, giving managers online access and enabling them to explore the features that interest them, such as performance gaps, time trends, or performance variations by region or organization.

#### System management

An administrator’s dashboard was added to the HIM to summarize the total number of systems connected to the HIM and the number of transactions successfully performed. An additional feature is the capacity to examine transactions that had errors or were not successfully performed. System administrators can use this feature to provide feedback to stakeholders on errors and improvement strategies, which helps to improve data quality.

## Results

Following the steps described above, Tanzania successfully completed the HIM implementation steps for all four use cases developed, enabling data exchange among 15 separate information management systems in the Tanzania HIS. The leadership team has completed five implementation steps for three of the business use cases defined by the leadership team: 1) client-level data exchange for priority hospitals; 2) aggregate data exchange for DHIS2; 3) health facility registry data extraction. As of this writing, the HIM is able to exchange data among the 14 systems.

### Community of practice helps with sustainability

In most LMIC’s, there is a paucity of talented technical personnel and within most Ministries of Health, this is the case. This is generally due to government salaries are considerably lower than private sector salaries; accordingly, highly talented technical staff are drawn away for 3–5 × salary increases. This means that staff turnover is moderate to high within most Ministries of Health. To address this shortfall of technical talent, the team “aggregated” technical talent from across the Government of Tanzania through the creation of a local community of practice (COP) with almost 25 participants meeting in person and virtually via WhatsApp and Skype. These participants learn together, work together, and support each other’s work. Some participating organizations had limited technical resources, both in number and in terms of their capacity. The COP enabled these organizations and facilities to share experienced technical staff among participating organizations towards a common goal. For instance, Mbeya Hospital’s technical resource provided technical support to staff at Kibong’oto Infectious Disease Hospital.

### Institutionalization of an HIE strategy

To help stakeholders consistently and systematically adhere to national standards, the HIE serves as the national reference guide to support data exchange among multiple health systems. Even as the MCSP Project began its close-out, the MOHCDGEC adopted the HIE as part of the national strategy for health care information, stipulating that any further investment in the system would follow the five-step process outlined above.

### Improved data quality and use

The HIM interoperability layer, as a core component in health information exchange, plays an important role in improving data quality and encouraging evidence-based planning and problem solving at all levels of the health system. For example, during implementation of the health data repository, the team realized that the data received at the HIE from participating organizations suffered from quality issues, such as data with future and historical dates, persons with discharge date prior to admission date, and reports of the same individuals dying in more than one ward. The interoperability layer’s filters captured these quality issues and requested rectification from the organization. The data quality problems captured by the layer also acted as a trigger for the organizations to further improve their own electronic systems.

### System and program benefits

Table 4 describes how the business cases changed: the challenges in these areas before implementation of the HIM, and system and programmatic benefits after implementation.

**Table 4:** Business use case, challenges, and outcomes after implementation of Health Information Mediator

Business use case	Challenge	Organizations	Sending systems	Receiving systems	System benefits of HIM	Programmatic benefits
Improving access and visualization of data from specialized hospitals	It was difficult to get data from large hospitals, since they do not report through DHIS2. There was a need to track the performance in these hospitals on a regular basis, looking at hospital performance indicators, such as, bed occupancy, services delivered, deaths occurring, and revenue collected	Ocean Road Cancer Institute	HINAYA	HDR and dashboard	Currently, 5 hospital EMRs electronically submit data to the HDR. It is a central client level data repository with records from all hospitals	Regular analysis of key indicators such as bed occupancy, deaths, services delivered, revenue collected, clients exempted and reimbursements from insurance. This is helpful in generating analytic insights on hospital performance
		Mirembe Mental Health Hospital	AfyaCare			
		Muungano Gateway Mbeya Zonal	Muungano Gateway			
		Referral Hospital Kibongoto	eMedical			
		Infectious Diseases Hospital	Care2x			
		Jakaya Kikwete Cardiac Institute	MEDPro			
		Muhimbili Orthopedic Institute	MEDPro			
		Muhimbili National Hospital	JEEVA			
Ability to analyze commodity data (eLMIS) alongside service delivery data (DHIS2)	No systematic analysis of services delivered (dhis2) and commodities consumed (eLMIS)	MOHCDGEC	eLMIS HRHIS	DHIS2	Automated data exchange from eLMIS to dhis2 every month by facility	Provides an analytical tool for managers to allow routine analysis by health facility and districts
Sharing key health facility details and status from the Health Facility Registry (HFR) with other information systems	Health facility details are constantly changing, such as, status from open to close or change in type, etc. All other systems need to have a list of all operational facilities to ensure supply and receive reports, etc. Every update had to be manually managed for all systems	MOHCDGEC	HFR	DHIS2, eLMIS, VIMS, HRHIS	One-to-many connection. The HFR only needs to be updated once and all updates to other systems are send electronically via the HIM	Health facility updates made in HFR are electronically sent to DHIS2, Vaccine Information Management System, electronic Logistics Management Information System, or eLMIS, and others
Facilitating the exchange of health commodities stock status from Medical Store Department (MSD) Epicor 9 to eLMIS	Epicor 9 and eLMIS are commodity management systems, however, it was difficult to see stock availability at MSD using Epicor 9 in eLMIS	MSD	E9	eLMIS		Managers can easily use eLMIS and see of commodities are available at MSD to fulfill requests submitted by health facilities

### Improved efficiency in data reporting and management

Improved data availability has been a key benefit of using automated data exchange. Before implementation of the mediator, the MOHCDGEC had no insights into hospital key performance indicators, such as bed occupancy, average length of stay, mortality by cause, revenue generated, or exemptions, because hospitals were not reporting them. Today, an average of 50,000 client records from among the five specialty hospitals are summarized electronically and sent to the HDR and dashboard every month. This has saved hospital staff the time needed to manually summarize these data each month, and also reduces the data quality errors that are bound to happen due to such a large number of transactions. Future implementation and scale-up of the mediator’s use will further reduce the time spent on manually summarizing client-level data, freeing more time to spend on further analysis and problem solving.

### Local ownership and capacity

MCSP closed its operations in Tanzania in June 2019, at the end of its agreement with USAID. The MOHCDGEC has the source codes of the mediator and trained staff who continue to maintain and manage the system. The ICT department continues to configure additional hospitals into the system and connect them to the mediator. The full system (the Health Data Mediator, Health Data Repository, Terminology Services, and dashboard with all software components) and all the data are hosted at the National Data Center in Tanzania; and Tanzanian in-country experts are solely responsible for managing, running, and scaling up the system. Limited external expertise will be required to provide handholding support for maintenance of the system and enhancing system performance and upgrades. In future, external support will be required to add additional use cases, such as HIV/AIDS case based surveillance, RMNCH continuum of care and tracking of clients, etc.

## Discussion

Improvements in data use should drive investments for building stronger interoperable health information systems. The steps described here enabled the MOHCDGEC and its partners to establish a strong base, including processes, guidance, and tools, for building interoperability across Tanzania’s health data systems.

Although there are still clear gaps in key components of Tanzania’s eHealth architecture (e.g., electronic medical records at lower levels of the health system, shared health records, master patient registry, etc.), the Ministry’s ICT unit is applying a stepwise approach to develop a comprehensive digital information system that aligns with the country’s broader e-Government policies and is guided by requirements and standards. Tanzania has planned implementation of multiple elements of the data exchange framework, and activities are already underway to link applications and automate data interchange. Data linkage and sharing will support greater transparency, inform policy- and decision-making, and improve client outcomes. Investment in technology over time allows the system to keep pace with innovative ways to collect, manage, and analyze data.^7,12^ Early cost–benefit analysis of some Tanzania upgrades to the HIS have shown a financial benefit, though additional benefits may not be seen for several years [18].

Future data exchange through the HIM mediator will further enhance the health system’s ability to share information, improve quality, and reduce duplication in three ways. *First*, it will facilitate access to data across the continuum of care—for instance, through the shared data repository, health workers can access historical data to check a woman’s history of high blood pressure during pregnancy. *Second*, interoperability improves integrated care and facilitates client tracking and referral from community to health facility. *Third*, data exchange between multiple domains will provide a wide range of data within a common repository—information about the patient and services provided; the provider and medicines/commodities provided; the service level (facility or community); and the payer and costs. This will be very useful source for quality and performance improvement initiatives.

Implementation of the HIM middleware was time-consuming, requiring connection of multiple heterogeneous systems and collaboration with multiple actors, all with competing priorities. Nonetheless, the demanding, continuous foundational activities that we completed during this initial phase not only paved the way not only for disparate systems to exchange data, but also provided a platform for building common understanding among all participants. Ongoing activities, since they build on previous accomplishments, promise to be faster and incur fewer delays compared to previous activities.

Working with third-party vendors also posed challenges. For example, it was difficult to convince offshore vendors to prioritize the system enhancements we needed to build integration and interoperability into the HIE. To obtain adequate engagement and support from the vendor’s side, it was critical to provide detailed information on the required steps for *each* use case as it affected each vendor’s product.

The first-phase activities described here represent a foundation, yet the system itself is still being developed. To sustain these gains and further amplify the use of the interoperability layer, the MOHCDGEC and its partners should continually add to the number of facilities and systems that use the interoperability layer to send data to the national health care repository. Also, developing a Shared Health Records file alongside a centralized Master Patient Index, as subcomponents of the Tanzania HIE, would enhance the exchange while maximizing the benefits of the interoperability layer. This could, for example, enable better visibility of anonymized patient-level data; enhance integrated and longitudinal care; increase citizen access to health information; enhance decision-making at the time of care, and enable clients to provide feedback on the care they received.

## Conclusions

High-income countries have made tremendous progress in interoperability, and continue to learn from and improving their systems. The U.S., for example, has seen major advances in the last 15 years, and states are supporting a range of initiatives across programmatic areas. Canada and European nations have also implemented integrated digital health programs. The concept of leveraging interoperability to improve health care has also gained significant notice in global and local public health.

A functioning, interoperable HIS is essential—not only to advance the WHO’s Universal Health Coverage agenda, but to ensure that every citizen receives quality care at an affordable cost. Advances made in developed countries, and the emerging global awareness of the importance of gathering and using quality data in health care, create a huge opportunity to share lessons globally and locally.

However, building the system described here takes time and requires collaboration of many partners and careful consideration of public health priorities; the architectural principles, hardware, and software options needed for interoperability; decisions about design structure; identification of critical gaps and priorities; and establishment of long-term partnerships with multiple committed organizations. A clear understanding that the time-consuming, iterative efforts to improve the system are balanced against the need to build capacity, provide health services, and maintain data collection until the new system is in place is also necessary.

The Government of Tanzania’s vision of a healthier population, its strong national health sector policy and eHealth strategy, and its long-term partnerships were all key ingredients in the successful development of the Tz-HIE. Countries that want to make their national health information systems more interoperable and sustainable would benefit from learning more about Tanzania’s HIE experience.

## Data Availability

All data generated or analysed during this study are included in this published article. The datasets generated during the current work are not publicly available as it contains personal identifiable information, but de-identified data may be available from the corresponding author on reasonable request.

## Abbreviations

ICT: Information, communication and technology
HIS: Health information systems
GAPS: Governance, architecture, program management, and standards
SDGs: Sustainable development goals
WHO: World health organization
EA: Enterprise architecture
MOHCDGEC: Ministry of health, community development, gender, elderly and children
Tz-HIE: Tanzania health information exchange
MCSP: Maternal and child survival program
USAID: U.S. agency for international development
DHIS2: District health information software
EMR: Electronic medical records
AeHIN: Asia eHealth information network
OpenHIE: Open health information exchange
GAPS-CU: Governance, architecture, program management, standards, capacity building and data use
PMO: Project management office
TWGs: Technical working groups
HSSP-IV: Fourth health sector strategic plan
eLMIS: Logistics management information system
MSD: Medical store department
HFR: Health facility registry
HIM: Health information mediator
HDR: Health data repository
CPT: Current procedure terminology

## Footnotes

1 https://digitalprinciples.org/.

2 Partners included the Tanzania University Computing Center (UCC), the Clinton Health Access Initiative (CHAI), PATH, the USAID funded Global Health Supply Chain Program, Management Sciences for Health, the U.S. Centers for Disease Control, and USAID/Washington and USAID/Tanzania.

3 Previously the Ministry of Health and Social Welfare.

4 https://broadbandcommission.org/Documents/publications/DigitalHealthReport2018.pdf.

5 Participating organizations are hospitals, clinics, or agencies that manage a HIS system. These were identified for the purpose of the implementation of the mediator. The MOHCDGEC is the overall agency in the health sector; however, hospitals or other agencies have their own management, leadership, rules, and regulations.

6 http://openhim.org/

7 https://www.openfn.org/.

8 https://www.health-e-link.net/.

9 https://www.mulesoft.com/.

## References

[1] Ministry of Health and Social Welfare. Health sector strategic plan III: July 2009—June 2015. In: Welfare TURoTMoHaS, ed.

[2] Mahundi M, Kaasboll J, Twaakyondo H. Health information systems integration in Tanzania: Tapping the contextual advantages. In: IST-Africa conference proceedings. 2011.

[3] Bakar NA, Selamat H. Investigating enterprise architecture implementation in public sector organisation: a case study of malaysia. Paper presented at: 3rd international conference on computer and information sciences; 2016, 2016.

[4] World health statistics 2017: monitoring health for the SDGs, Sustainable Development Goals*.* Geneva: World Health Organization;2017. https://www.who.int/gho/publications/world_health_statistics/2017/EN_WHS2017_TOC.pdf?ua=1.

[5] World Health Organization. Monitoring the building blocks of health systems: a handbook of indicators and their measurement strategies*.* Geneva: WHO; 2010 2010. http://www.who.int/healthinfo/systems/WHO_MBHSS_2010_cover_toc_web.pdf?ua=1.

[6] Kimaro HC. Strategies for developing human resource capacity to support sustainability of ICT based health information systems: a case study from Tanzania. The Electronic Journal of Information Systems in Developing Countries*.* 2006;26(0). http://www.ejisdc.org/ojs2./index.php/ejisdc/article/view/272.

[7] Adenuga OA, Kekwaletswe RM, Coleman A (2015). eHealth integration and interoperability issues: towards a solution through enterprise architecture. Health Inf Sci Syst..

[8] Mudaly T, Moodley D, Pillay A, Seebregts CJ. architectural frameworks for developing national health information systems in low and middle income countries. Paper presented at: Enterprise Systems Conference; 2013/11//, 2013.

[9] Maokola W, Willey BA, Shirima K (2011). Enhancing the routine health information system in rural southern Tanzania: successes, challenges and lessons learned. Trop Med Int Health.

[10] Rumisha SF, Mboera LE, Senkoro KP, Gueye D, Mmbuji PK (2007). Monitoring and evaluation of integrated disease surveillance and response in selected districts in Tanzania. Tanzan Health Res Bull.

[11] Ishijima H, Mapunda M, Mndeme M, Sukums F, Mlay VS (2015). Challenges and opportunities for effective adoption of HRH information systems in developing countries: national rollout of HRHIS and TIIS in Tanzania. Hum Resour Health.

[12] Braa J, Kanter AS, Lesh N, et al. Comprehensive yet scalable health information systems for low resource settings: a collaborative effort in Sierra Leone. AMIA Annu Symp Proc*.* 2010;2010:372–376. http://www.ncbi.nlm.nih.gov/pmc/articles/PMC3041283/. PMC3041283 21347003

[13] Braa J, Heywood A, Sahay S (2012). Improving quality and use of data through data-use workshops: Zanzibar, United Republic of Tanzania. Bull World Health Organ.

[14] Rusu L, Tenga RP (2010). IT governance in the healthcare sector: a case study of a public and private hospital in Tanzania. Int J Inf Syst Change Manage.

[15] Ministry of Health; Community development; gender; elderly and children. Health Sector Strategic Plan 2015–2020 (HSSP IV).

[16] USAID|DELIVER PROJECT. USAID|DELIVER PROJECT Final Country Report: Tanzania Arlington, VA: USAID | DELIVER PROJECT, Task Order 4;2016. http://deliver.jsi.com/wp-content/uploads/2016/12/FinaCounRepo_TZ.pdf.

[17] Supply Chain Management System, USAID|DELIVER PROJECT Task Order 4, 7 UDPTO. Health logistics in Tanzania: a decade of supply chain accomplishments for supply chain interventions*.* Arlington, VA: USAID | DELIVER PROJECT Task Order 4, USAID | DELIVER PROJECT Task Order 7 2016. https://www.jsi.com/JSIInternet/Inc/Common/_download_pub.cfm?id=17395&lid=3.

[18] Mwencha M, Rosen JE, Spisak C, Watson N, Kisoka N, Mberesero H (2017). Upgrading supply chain management systems to improve availability of medicines in Tanzania: evaluation of performance and cost effects. Glob Health, Sci Pract.

[19] Leon N, Schneider H, Daviaud E (2012). Applying a framework for assessing the health system challenges to scaling up mHealth in South Africa. BMC Med Inform Decis Mak.

[20] Bediang G (2010). Medical decision support systems in Africa. Yearb Med Inform.

[21] Dawes RC (2003). Investigating the interface between health system reform and HIV/AIDS in sub-Saharan Africa: an approach for improving the fight against the epidemic. Afr J AIDS Res..

[22] Gichoya J, Pearce C, Wickramasinghe N (2013). Information architecture considerations in designing a comprehensive tuberculosis enterprise system in Western Kenya. Stud Health Technol Inform.

[23] Armstrong CE, Magoma M, Ronsmans C (2015). Magnitude of maternal and neonatal mortality in tanzania: a systematic review. Int J Gynaecol Obstet.

[24] Atun R, Weil DEC, Eang MT, Mwakyusa D (2010). Health-system strengthening and tuberculosis control. Lancet.

[25] Pemba S, Macfarlane SB, Mpembeni R, Goodell AJ, Kaaya EE (2012). Tracking university graduates in the workforce: information to improve education and health systems in Tanzania. J Public Health Policy.

[26] Kumalija CJ, Perera S, Masanja H (2015). Regional differences in intervention coverage and health system strength in Tanzania. PLoS One..

[27] Ministry of Health; Community Development; Gender; Elderly and Children. Tanzania health information exchange project charter: an implementing document for the ehealth strategy. In: the united republic of tanzania ministry of health; community development; gender; elderly and children, 2015.

[28] President's Office. e-government related standards and guidelines. In: President’s Office PSMae-GA, ed. Dar es Salaam2015.

[29] PwC. e-Government related Standards and Guidelines, Operational Manual. Dar es Salaam: President’s Office, Public Service Management and e-Government Agency, 2015.

[30] Winter R, Schlep J. Enterprise architecture governance: the need for a business-to-IT approach. Paper presented at: Proceedings of the 2008 ACM symposium on Applied Computing 2008.

[31] Higman S, Dwivedi V, Nsaghurwe A, Busiga M, Sotter Rulagirwa H, Smith D, Wright C, Nyinondi S, Nyella E (2018). Designing interoperable health information systems using enterprise architecture approach in resource-limited countries: a literature review. Int J Health Plann Manage.

[32] Mensah Abrampah N, Syed SB, Hirschhorn LR (2018). Quality improvement and emerging global health priorities. Int J Qual Health Care..

[33] Bernard S. Introduction to enterprise architecture: linking strategy, business and technology*.* Third ed: AuthorHouse; 2012.

[34] Evernden R, Evernden E. Enterprise architecture - the eight fundamental factors: a practical guide to the eight fundamental factors that are common to all EA approaches and frameworks*.* 2nd ed: CreateSpace Independent Publishing Platform; 2015.

[35] Mwanyika H, Lubinski D, Anderson R, et al. Rational systems design for health information systems in low-income countries: an enterprise architecture approach. J Enterprise Archit*.* 2011:60–69.

[36] Virkanen H, Mykkänen J (2014). Analysis of central enterprise architecture elements in models of six eHealth projects. Stud Health Technol Inform.

[37] Maternal and Child Survival Project. Tanzania country summary*.* 2017. https://www.mcsprogram.org/wp-content/uploads/2017/04/Tanzania-Country-Summary-March-2017-1.pdf.

[38] Ministry of Health CD, Gender, elderly and children. Immunization and vaccine development programme, 2016–2020 comprehensive multi year plan*.* 2016. http://www.moh.go.tz/sw/mpango-wa-taifa-wa-chanjo?download=238:comprehensive-multiyear-plan-2016-%E2%80%93-2020

[39] Data Use Partnership: The Journey to Better Data for Better Health in Tanzania, 2017 - 202*3.* Ministry of Health, Community Development, Gender, Elderly, and Children, PATH; 2016. https://path.azureedge.net/media/documents/DHS_health_tanzania_rpt1.pdf

[40] Lungo JH (2008). The reliability and usability of district health information software: case studies from Tanzania. Tanzan J Health Res.

[41] Madon S, Amaguru JO, Malecela MN, Michael E (2014). Can mobile phones help control neglected tropical diseases? Experiences from Tanzania. Soc Sci Med.

[42] Maokola W, Willey BA, Shirima K (2011). Enhancing the routine health information system in rural southern Tanzania: successes, challenges and lessons learned. Trop Med Int Health TM & IH.

[43] Atun R, de Jongh TE, Secci FV, Ohiri K, Adeyi O, Car J (2011). Integration of priority population, health and nutrition interventions into health systems: systematic review. BMC Public Health.

[44] Principles for Digital Development. https://digitalprinciples.org/principles/. Accessed 6/10/2019.

[45] Mikkelsen-Lopez I, Shango W, Barrington J, Ziegler R, Smith T, deSavigny D (2014). The challenge to avoid anti-malarial medicine stock-outs in an era of funding partners: the case of Tanzania. Malar J.

